# Intra- and Juxta-Articular Osteoid Osteoma Mimicking Arthritis: Case Series and Literature Review

**DOI:** 10.3390/children10050829

**Published:** 2023-05-02

**Authors:** Adele Civino, Federico Diomeda, Luca Giordano, Maria Beatrice Damasio, Sandra Perrone, Romina Gallizzi, Angelo Ravelli, Prisco Piscitelli, Maria Cristina Maggio

**Affiliations:** 1 Pediatric Rheumatology and Immunology Unit, “Vito Fazzi” Hospital, 73100 Lecce, Italy; federicodiomeda91@gmail.com; 2 Department of Radiology, “Vito Fazzi” Hospital, 73100 Lecce, Italy; 3 Department of Radiology, IRCCS Istituto Giannina Gaslini, 16147 Genova, Italy; 4 Department of Radiology, “Cardinale G. Panico” Hospital, 73039 Tricase, Italy; 5 Pediatric Unit, Department of Health Science, Magna Grecia University of Catanzaro, 88100 Catanzaro, Italy; 6 Scientific Direction, IRCCS Istituto Giannina Gaslini, 16147 Genova, Italy; 7 Department of Neurosciences, Rehabilitation, Ophthalmology, Genetics and Maternal-Infantile Sciences (DiNOGMI), University of Genova, 16147 Genova, Italy; 8 Department of Biological and Environmental Sciences and Biotechnologies, University of Salento, 73100 Lecce, Italy; 9 Health Authority, ASL LE, 73100 Lecce, Italy; 10 Department Pro.Sa.M.I., University of Palermo, 90133 Palermo, Italy

**Keywords:** osteoid osteoma, arthritis, child, joint pain, synovitis, juvenile idiopathic arthritis, rheumatic diseases, bone tumor, imaging, diagnosis

## Abstract

Background: Intra- and juxta-articular osteoid osteomas are rare, representing less than 10% of all osteomas. Compared to the classic diaphyseal or metaphyseal site of long bones, they often have an atypical onset, a longest diagnostic delay, and frequent initial misdiagnoses, with pictures that can mimic inflammatory monoarthritis. We aimed to describe a case series, and to provide a literature review of this uncommon and misleading tumor location. Methods: We performed a retrospective analysis of patients referred to three pediatric rheumatology centers, with a final diagnosis of articular osteoid osteoma. A review of the literature was additionally conducted. Results: We included 10 patients with a mean age of 14 years. All patients with unusual sites (olecranon fossa, lumbar vertebra, distal phalanx of the toe, fibula) had a misdiagnosis, and cases with initial suspicion of monoarthritis had the longest diagnostic delay, up to 24 months. The literature review confirms the significant risk of misdiagnosis, and an average time from symptom onset to diagnosis ranging from 0.4 to 1.8 years. Conclusions: Articular osteoid osteoma may mimic arthritis, especially in adolescence. Knowledge of the atypical forms of presentation, and of the clinical and radiological pitfalls, reduces the risk of diagnostic error.

## 1. Introduction

Osteoid osteoma (OO), a common benign bone tumor, was first recognized as a clinical entity by Jaffe in 1935 [1]. It represents approximately 10% of all benign skeletal lesions, and is most frequently seen in adolescents and young adults, affecting males three times more commonly than females [2,3]. The most frequent location is the diaphysis or metaphysis of the long bones, often the femur, but any other site may be involved [3]. The lesion is an active bone nidus within a highly vascularized stroma, surrounded by reactive sclerotic bone tissue. The nidus tends to expand in volume beyond 2 cm, and remodeling of the bone occurs through osteoblastic and osteoclastic activity, with some spontaneous regression reported [4]. The nidus matrix and surrounding reactive zone are generally well-innervated by peripheral sensory nerve fibers, and produce high levels of prostaglandins, which mediate vasodilation and inflammation in the surrounding tissues, causing pain that is typically worse at night and improves with nonsteroidal anti-inflammatory drugs (NSAIDs) [5,6,7]. Unusual location and an atypical clinical presentation may mimic other diseases, especially in the intra- or juxta-articular OOs, estimated to comprise 5.2–10% of all OOs [3,8,9,10]. The most frequent misdiagnoses reported in small case series or case reports of intra-articular OOs in children and young adults include juvenile idiopathic arthritis (JIA), juvenile spondyloarthritis, hip synovitis, complex region pain syndrome, recurrent multifocal osteomyelitis, infection, traumatic lesion, “growing pains”, soft tissue tumor, and hip impingement syndrome [9,11,12,13,14]. A rheumatic misdiagnosis might lead to diagnostic delay and inappropriate therapy, such as intra-articular corticosteroid injection or immunosuppressive drugs [15,16,17].

Data remain scarce on the presenting features of articular OOs in pediatric patients and the most useful clues that can guide the correct diagnosis.

In view of this information, we aimed to describe a multicenter retrospective pediatric case series of OOs initially referred to pediatric rheumatology centers, and to review published data on intra- or juxta-articular OOs in children, highlighting the features useful for differential diagnosis.

## 2. Materials and Methods

We collected retrospective data on pediatric patients (≤18 years) with a final diagnosis of intra- or juxta-articular OOs, referred to the pediatric rheumatology centers of Lecce, Genoa, and Palermo in Italy, between 2010 and 2022. All cases required written informed consent. We obtained the following demographic and clinical information from available medical records: sex, age at diagnosis, OO location, presenting symptoms, pain features (night pain, i.e., pain that worsens at night, pain with activity, relief with NSAIDs), specific joint involved, abnormal range of joint motion, joint swelling, morning stiffness, initial misdiagnosis, time to diagnosis, imaging, histopathology, and definitive treatment.

To obtain more data on the topic, we performed a literature review to identify articles of potential interest about OOs with joint involvement in children and adolescents. We searched PubMed to find relevant articles published in English, with no restrictions on publication year, up to 1 February 2023. The following search string was used: “Osteoid osteoma” [Title/Abstract] AND (joint [Title/Abstract] OR articular [Title/Abstract] OR intra-articular [Title/Abstract]). We also reviewed references from relevant articles not identified in the original search.

We included articles in this review if they comprised case series or case-control studies, including patients with a definitive diagnosis of OO, aged 0–18 years. We excluded articles in which demographic and/or clinical aspects of our reference age range could not be distinguished from adults.

## 3. Results

### 3.1. Case Series

We included 10 patients with intra- or juxta-articular OOs, with a mean age of 14 years (range 5–18) [Table 1].

Seven patients were female and three were male. The localization of the lesions was as follows: three in the femoral neck, two in the upper tibia, one in the acetabulum, one in the olecranon fossa of the humerus, one in the distal toe phalanx, one in the distal fibula, and one in the vertebral pedicle L1. Joint pain or, less frequently, back pain was the presenting complaint, with the hip (4/10) and knee (3/10) most often involved. The restricted joint motion was associated with pain in 7/10 patients. None of the patients had morning stiffness.

The majority of patients (7/10) reported nocturnal pain, and 6 of them had the typical response to aspirin or NSAID. Seven of the 10 patients received an initial misdiagnosis: monoarticular arthritis (4), ileo-psoas enthesopathy (1), amplification pain syndrome (1), and traumatic vertebral fracture (1).

**Figure 1:**
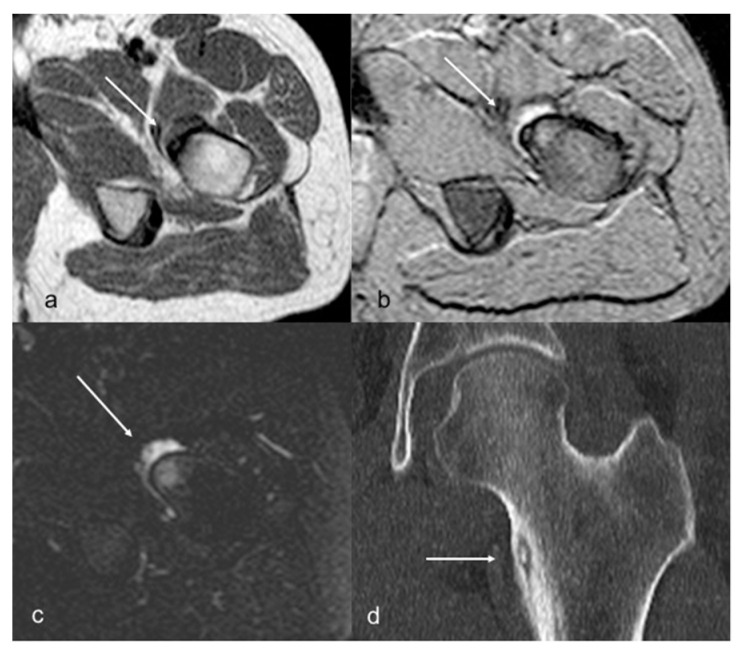
Patient 3. Axial T1 TSE (**a**), T2 FFE (**b**), and STIR (**c**) MR images showing a small cortical nidus (arrows) within the femur shaft, with a target-like appearance, sclerosis, bone marrow edema, and synovial effusion. CT (**d**) better detected the partially mineralized nidus, cortical thickening, and sclerosis than did MRI.

**Figure 2:**
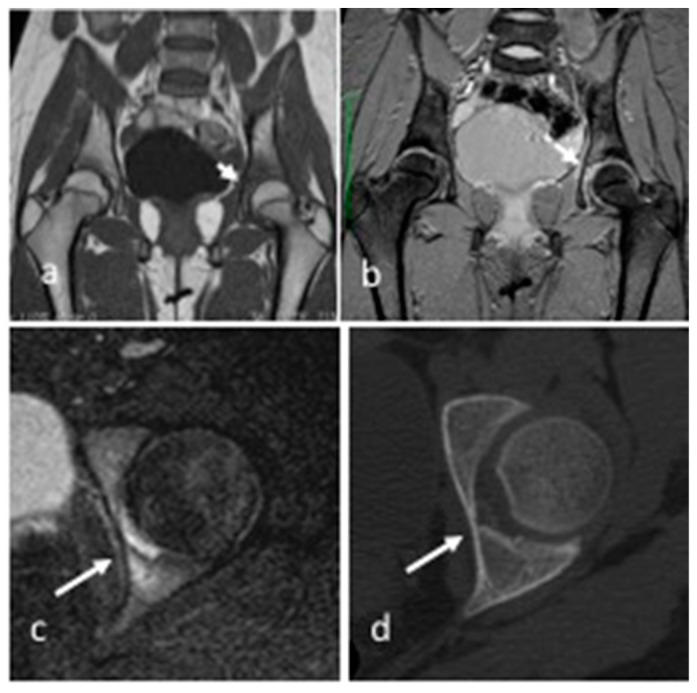
Patient 4. Coronal T1 TSE (**a**), T2 FFE (**b**), and axial STIR (**c**) MR images showing a small subchondral lesion (arrows) hyperintense in T2 on the upper acetabular profile of left hip, with perilesional sclerosis, bone marrow edema, and reactive synovitis. CT (**d**) better detected the small lytic lesion with a sclerotic edge overflowing from the upper profile of the acetabulum.

**Figure 3:**
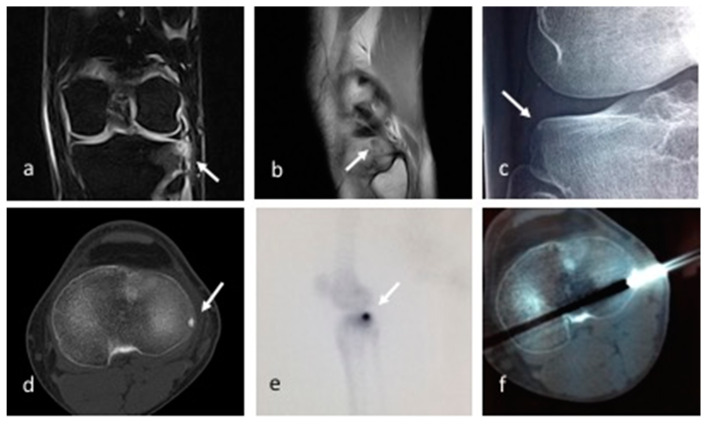
Patient 6. Coronal STIR (**a**), sagittal T1 MR images (**b**) showing a small subcortical nidus (arrows) within the external tibial plateau with a target-like appearance, bone marrow edema, and reactive synovitis. X-ray (**c**) and CT (**d**) better detected the small lytic lesion with partially mineralized nidus. CT percutaneous biopsy and radiofrequency ablation (**f**) were performed. Bone scintigraphy with technetium-99: the lesion is represented by a central nidus with very high uptake, surrounded by a larger area with moderate activity, consisting of the double-density sign (**e**).

**Figure 4:**
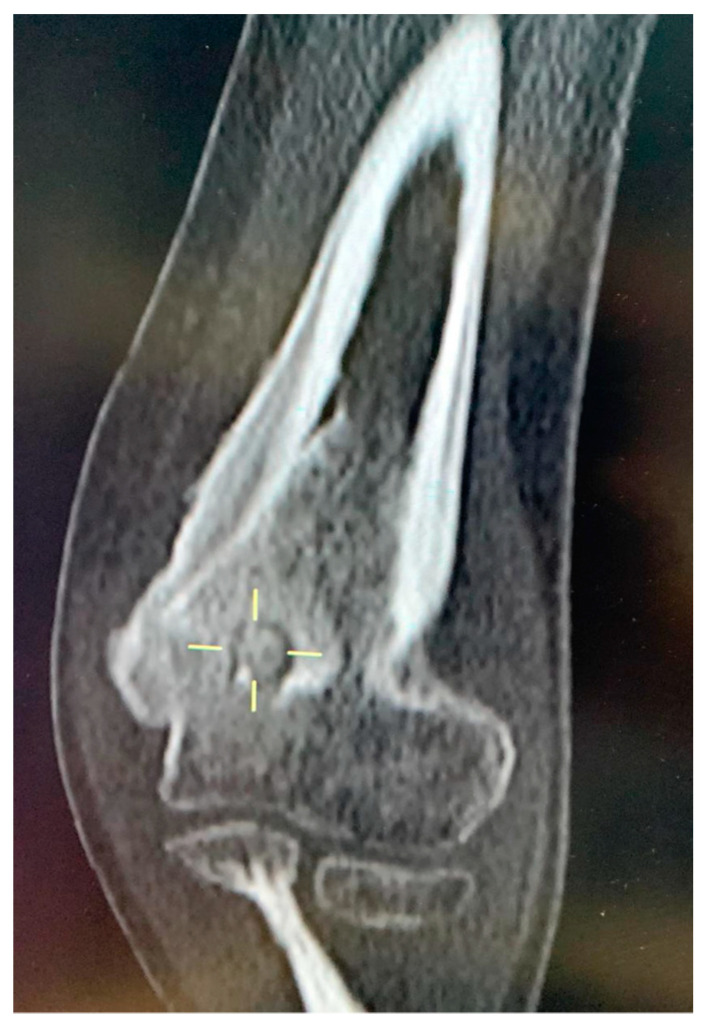
Patient 8. Volumetric CT of the elbow: Coronal reconstruction, bone windowing. Focal rounded hyperdense lesion with hypodense peripheral ring within the olecranon fossa suggestive of a “nidus” (7 mm diameter). Osteothickener trabecular pattern of the surrounding bone, with focal thickening of the distal meta-diaphyseal humeral cortex, more evident on its ulnar side.

**Figure 5:**
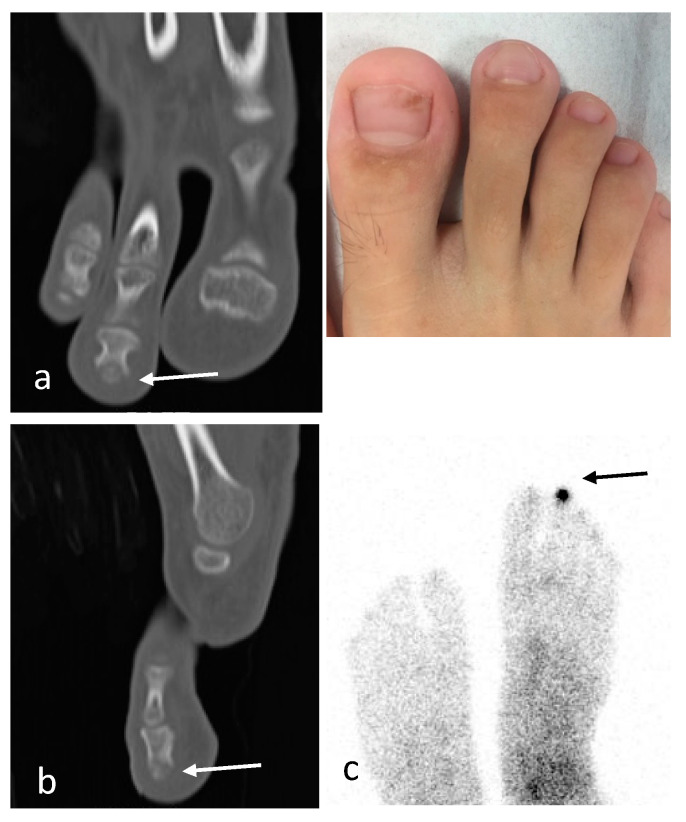
Patient 9. (**a**,**b**) CT detected the small lytic lesion with partially mineralized nidus (white arrows) in the nail end of the distal phalanx of the second ray. (**c**) Bone scintigraphy with technetium-99 showing a central nidus with very high uptake (black arrow).

**Figure 6:**
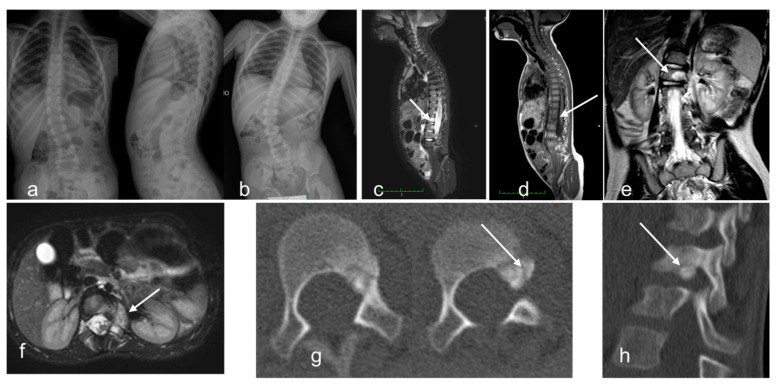
Patient 10. Rx spine in orthostatism. 20 days after the trauma: scoliosis with wide radius dorso-lumbar right-convex (**a**). 40 days after the trauma: patient suffering from intense pain, unable to keep the station upright. Aggravation of scoliosis with lumbar fulcrum (**b**). Sagittal STIR (**c**) and coronal T2 TSE (**e**) MR images showing somatic and peduncle bone marrow edema (arrows). Axial STIR MR images showing secondary thickening of paravertebral soft tissues (**f**). Sagittal T1 TSE MR images showing suspected fracture with bone callus (**d**). CT better detected a small 9 mm round expansive formation between L1 vertebral soma and left peduncle (**g**). The lesion protrudes into the conjugation canal, and it is in continuity with bone tissue thickened (**h**). Typical osteoid osteoma.

Plain radiographs were available for 7 patients, with no abnormalities in three cases, a periosteal reaction in two cases, worsening scoliosis in one case, and a typical radiolucent nidus within the external tibial plateau in one patient. Joint effusion was detected by imaging in all patients, except for of two cases, localized, respectively, in the distal fibula and the vertebral pedicle L1. MRI was performed in 6 patients, with findings suggestive of osteoma in three cases, and nonspecific changes in the remaining half of the cases. CT was used to confirm the diagnosis in all patients, with the identification of the typical nidus in all the cases [Figure 1, Figure 2, Figure 3, Figure 4, Figure 5 and Figure 6]. Final treatment data were available for five patients: three underwent CT-guided RF ablation, and two underwent surgical resection.

The average time between the onset of symptoms and the correct diagnosis of OO was 7 months (range 1–12). Three patients with an initial diagnosis of inflammatory monoarthritis had the longest time to diagnosis, respectively, 9, 12, and 24 months.

The first case was a 14-year-old girl, who initially presented to the orthopedist with elbow swelling, pain, and limitation of motion, attributed to a recent sprain. She was treated with cast immobilization for two weeks, without improvement, and, one month later, MRI showed joint effusion and bone marrow edema of the capit radium. Two months after the trauma, she was revaluated by an orthopedist, who suspected a strain of the medial collateral ligament. The girl presented partial pain relief with NSAID therapy, without improvement of the joint swelling, and she was hospitalized in a local pediatric unit.

Hemogram and C-reactive protein were normal, erythrocyte sedimentation rate slightly increased (25 mm per first hour), anti-nuclear antibodies (ANA), anti-cyclic citrullinated peptide antibody, and rheumatoid factor were all normal. X-ray showed a periosteal reaction in humeral metaphysis, and MRI confirmed joint effusion and synovial hypertrophy, with enhanced synovial contrast after gadolinium administration. Synovial fluid analysis demonstrated an inflammatory response, with a prevalence of lymphocytes and monocytes; synovial biopsy demonstrated non-specific mononuclear infiltration. Six months after the trauma, she was referred to a tertiary pediatric rheumatology center. In the suspicion of post-traumatic inflammatory reactive arthritis, she was treated with NSAIDs and steroid joint injection with triamcinolone hexacetonide, with a transient resolution of joint swelling and limitation of motion, but persistence of pain. Repeated investigations showed a periosteal reaction of the humeral diaphysis on x-ray, and a CT scan showed a typical nidus located in the olecranon fossa. The diagnosis of OO was confirmed by histology, 9 months after the onset of symptoms.

The second case with a delayed diagnosis was a 15-year-old boy presenting to an immunologist, with a two-month history of painful swelling of the distal phalanx of the right second toe. All laboratory tests, including blood cell count, acute phase reactants, and ANA, were normal. He was initially treated with oral steroid, without benefit, then, with naproxen, with improvement of pain. Nine months after the onset of symptoms, he was referred to an adult rheumatologist for persistent swelling and pain, partially controlled by NSAIDs. Ultrasonography showed a subungual hypoechoic area with positive Doppler signal interpreted as enthesitis, with normal x-ray and minimal joint effusion of the distal interphalangeal joint, with subcutaneous edematous changes on MRI. Psoriatic juvenile idiopathic arthritis was suspected by the presence of a first-degree relative with psoriasis and subungual enthesitis with arthritis of the distal interphalangeal joint. The boy was sent to a pediatric rheumatology center one year after the onset of symptoms, but the clinical features, with nocturnal intensifying pain and rapid responsiveness to NSAIDs led to the suspicion of osteoma, confirmed by CT scan and histology.

Finally, the longest time to diagnosis was recorded in a 10-year-old girl, referred to the pediatric rheumatology center for a recurrent, unexplained limp that started two years earlier. The primary care pediatrician initially performed blood cell counts, acute phase reactants, ANA, hip ultrasound, and x-ray, all of which were normal, and suspected a recurrent transient hip synovitis, favored by excess body weight. At the first evaluation in the pediatric rheumatology center, she reported episodic mild nocturnal pain in the right groin, with radiation to the knee, that resolved spontaneously. The examination revealed limitation of hip motion, especially the abduction and internal rotation. MRI and CT showed a calcified nidus located in the right femoral neck.

### 3.2. Literature review

The search terms were matched by 656 references. We selected case series or case-control studies including patients aged 0–18 years with a definitive diagnosis of OO, defining a group of 22 articles. We then excluded articles in which demographic and/or clinical aspects of our reference age range could not be distinguished from adults, obtaining the final group of 13 studies included in this review, with a total of 147 patients (52% male) aged 0–18 years, diagnosed with intra- or juxta-articular OOs. Most of the studies included pediatric and adult patients [3,8,15,17,18,19,20,21], while only 5 focused exclusively on the pediatric age [16,22,23,24,25] (Table 2).

The studies of Goldberg et al. and May et al. included the largest number of pediatric patients, 31 and 50, respectively [22,23].

Five studies described patients with specific joint involvement, namely, the hip [22,23], elbow [15,17], or ankle [20]. Two studies on pediatric patients found the hip joint to be the most common location [4,26]. We evaluated the distribution of OO locations, excluding cases series that included patients with a specific joint involved. The hip was confirmed as the most frequently involved joint in 34/53 (64%) patients (femur 25, acetabulum 4, not specified 5), followed by the ankle in 9/53 (17%) (talus 7, calcaneus 2), elbow 2, knee 2, shoulder 2, little foot joint 2, wrist 1, lumbar spine 1.

The average age at diagnosis was equal to or higher than ten years in all the studies; nevertheless, children as young as two years old have been reported, such as in the hip cohorts of Goldberg et al. [22]. Considering articles with complete demographic data, patients with intra-articular OOs younger than 10 years were 7/46 (15%).

Pain was reported as the presenting symptom in all patients, while other symptoms related to joint involvement were not always collected. We calculated the frequency of joint symptoms and specific pain features, considering patients for whom data were available: pain alleviated by NSAIDs was present in 98/132 (74%), nocturnal pain in 80/119 (67%), limitation of joint motion (LOM) in 76/129 (59%), pain with activities in 29/54 (53%), joint swelling in 6/23 patients (26%).

All but two of the studies reported a mean duration of symptoms greater than 1 year (range 0.4–1.8 years). Considering articles with complete data on the initial diagnostic suspicious, we found a first misdiagnosis in 66/109 patients (60%). A specific misdiagnosis of arthritis was evaluated in 8/13 articles, with a reported prevalence of 23/118 pts (19%). One study, a case series of 4 patients of Traore et al., focused specifically on pediatric intra-articular OOs misdiagnosed as JIA and, initially, inappropriately treated with intra-articular corticoid injection [16].

Finally, we collected data on the definitive treatment of 122 patients, excluding articles specifically focused on a treatment regimen: 63/122 (51%) underwent surgical excision (curettage, en block excision), 50/122 (40%) radiofrequency ablation (CT guided, arthroscopic), and 9/122 (7%) conservative treatment.

## 4. Discussion

Osteoid osteoma is typically located in the diaphysis or metaphysis of long bones, and, in many cases, the diagnostic suspicion is facilitated by the presence of typical bone pain that worsens at night with an excellent response to NSAIDs. However, the diagnosis can be challenging when the lesion is located in the joint or the periarticular region, with a clinical picture that can mimic inflammatory arthritis.

This is, to the best of our knowledge, the first review focusing on pediatric cases of intra- or juxta-articular OOs, as well as the largest case series from the perspective of pediatric rheumatologists.

We retrospectively collected the clinical picture and imaging findings of 10 children and adolescents initially referred to three pediatric rheumatology centers for joint or back complaints, with a final diagnosis of intra- or juxta-articular OOs. The lesion was within the joint capsule in half of the cases, and the nidus was intracortical in most of our patients. An OO can be defined as intra-articular, if the nidus is within the joint capsule, while the definition of juxta-articular OOs is more applicable for tumors laying in the proximity of a joint giving articular symptoms [27]. Otherwise, differentiation between juxta-articular and complete extra-articular is difficult, as in the case of OO of the foot and ankle. Here, the distance between an OO located in the center of a small bone or a periarticular area is just speculative [20]. Song et al. demonstrated, in their comparison between intra- and extra-articular OOs, that, in intra-articular OOs, all the niduses were located in the cortex, while, in extra-articular OOs, niduses were located at different levels: cortex, medullary canal, and subperiosteal region [25].

The hip was the most frequent localization in our patients, and the literature review confirmed this data, as the most affected joints were the hip (64%) and ankle (17%). According to the largest cohorts of hip OOs, lesions are rarely found in the acetabulum, and are more commonly found in the proximal femur [22,23]. Some periarticular sites are very rare and are associated with diagnostic pitfalls. OO of the phalangeal bones may be associated with marked soft tissue swelling, mimicking dactylitis, sausage-like tender swelling of one or more digits extending beyond the joint margin [28]. This clinical feature can lead to a misdiagnosis of juvenile spondylarthritis, as in our case, in which the presence of a suspected dactylitis of the second toe, together with psoriasis in a first-degree relative, led to an initial diagnosis of psoriatic arthritis. Localization in the distal phalanx is associated with a typical enlargement of the nail, and painless swellings are often reported, due to the absence of intralesional nerve fibers. The vertebral localization of the OO generally affects the lumbar tract and the posterior elements: the laminae, facet joint, or pedicle. It is commonly associated with pain-related scoliosis and muscle spasms, misdiagnosed in more than half of the cases [29]. Our case had worsening painful scoliosis after a trauma, with initial suspicion of L1 pedicle fracture, and diagnosis of osteoid osteoma, identified by CT scan.

In articular osteomas, not only may the location be unusual and difficult to diagnose, but also the pain characteristics may be less suggestive. Bone pain worsening at night is a common feature in OOs. Although less frequently, this symptom can represent a red flag for pediatric malignancies, being reported in 20% of solid malignancies presenting with musculoskeletal symptoms [30]. The most typical diagnostic clue of OO is the association of intermittent pain that becomes worse at night and is relieved by salicylates or NSAIDs. Song et al. reported that this feature was less frequent in children with intra-articular OO (36.4%) than in children with extra-articular OO (69.6%), and the chance of misdiagnosis and the diagnostic delay was significantly higher in intra-articular OO [8,25]. More than half of patients had an initial misdiagnosis in our review, with suspicion of arthritis in 20% of patients.

Another factor to consider in the presentation of articular osteoma is the possibility of referred pain in sites other than the location of the tumor. The most frequent cases reported in the literature regard hip OOs with referred pain to the knee, but sacroiliac OOs with referred pain to the hip have also been reported [19,21,31]. In our cases, the patient with the longest time to diagnosis was a 10-year-old girl with a final diagnosis of right femoral neck OO, that presented with recurrent pain in the right groin with radiation to the knee, lasting two years.

In addition to the lower frequency of the typical clinical picture, the presence of signs of joint inflammation as leading symptoms also points toward other causes. In fact, intra-articular OO may cause a synovial reaction, with proliferation and chronic nonspecific synovitis, favored by the high levels of prostaglandins, which mediate vasodilation, inflammation, and pain [6,7]. Frequently, physical examination reveals LOM, present in more than half of cases in the literature review, and in almost all cases of histologically confirmed intra-articular osteoma [8]. Persistent joint pain associated with LOM, or the joint swelling or synovial effusion revealed by imaging with unknown cause, may lead to the misdiagnosis of JIA monoarthritis. Features atypical for JIA include isolated hip involvement at onset, common in osteoma, absence of morning stiffness, and poor response to conventional therapies [16,30]. Failure to respond to joint steroid injection, as observed in our case with elbow arthritis, requires careful radiological re-evaluation.

In two of our three cases with an initial diagnosis of inflammatory arthritis, the features that influenced this suspicion were the persistence of swelling with joint effusion documented by imaging, with inflammatory signs (positive power Doppler signal, synovial hyperplasia with contrast enhancement), and nonspecific findings on x-ray. In both cases, the presence of nocturnal pain and the partial response to NSAIDs could guide towards the correct diagnosis.

Another diagnostic challenge of intra- or juxta-articular localization of OOs is the lower frequency of typical radiological findings. Identification with standard x-ray is often very difficult, due to the low frequency of periosteal reaction, present in only 1/3 of our case series. The computed tomography (CT) scan is the most useful diagnostic method for identifying the nidus of an OO. Even in the absence of a sclerotic rim, a CT scan usually shows a distinct separation between the nidus and the surrounding normal trabecular bone. The role of magnetic resonance imaging (MRI) in the diagnosis of osteoid osteoma is debatable [8]. MRI may, in fact, neglect the nidus, depending on tumor location, involvement of small bones, and the severity of the surrounding bone inflammatory reaction [32,33]. Today, diagnosis is reached virtually in 100% of patients with CT, as confirmed by most recent literature [22]. MRI is often preferred over CT in suspected inflammatory arthritis, and this may lead to a delay in the diagnosis of osteoid osteoma.

The various clinical and radiological diagnostic pitfalls make diagnosis a challenge and lead to a significant diagnostic delay. In fact, in most studies, the average time to reach a correct diagnosis in pediatric patients with intra- or juxta-articular OOs is between 1 and 2 years, while time to diagnosis is less than 1 year in patients with extra-articular sites [8,25].

Although some cases of spontaneous regression were reported, the definitive treatment of OOs is complete lesion ablation. Traditionally, open excision to remove the nidus has been the gold standard in OO treatment [34]. Percutaneous CT-guided mechanical or thermal ablation of the nidus is a modern approach that has been made possible by the advancement of imaging modalities [35]. Radiofrequency ablation (RFA) is a technique that has spread over the last 20 years, becoming popular, and minimizing the complications associated with more extensive surgery [36]. An arthroscopic approach has recently been proposed for OOs located at the intra-articular bony surfaces [37]. Skeletal abnormalities, such as coxa valga deformities or leg length discrepancies, are associated with intra-articular localization of OOs, but this did not always appear to be clinically relevant [25]. It is unclear whether the tumor or the treatment may be the primary cause of these complications [23].

Some caveats should be considered in our study, apart from its retrospective nature. The small sample size of our case series and the heterogeneity of the joints involved do not allow an analysis of the predictive factors of the correct diagnosis. For this reason, we focused on the description of some pitfalls of the cases with the longest diagnostic delay, which may be useful for future analysis on larger series. In the literature, most studies involve heterogeneous data of juxta-articular and intra-articular OOs, and only a few articles focused exclusively on children. A multicenter prospective study, focused on juxta or intra-articular OOs in children, could better identify clinical and radiological features, and the best diagnostic and therapeutic options. We also recognize the importance of defining a more comprehensive picture of all benign or malignant periarticular tumors that may mimic arthritis in children.

## 5. Conclusions

Intra- or juxta-articular OOs are characterized by a significant risk of diagnostic error and delay, often exceeding one year from the onset of symptoms. Careful clinical history and imaging review are necessary to not miss a diagnosis, keeping in mind some diagnostic pitfalls, such as joint inflammation as a leading symptom, an atypical pattern of pain, and lower frequency of specific radiological findings. Performing a CT scan in suspected cases leads to the correct diagnosis and avoids prolonged symptoms and inappropriate therapies.

## Figures and Tables

**Table 1:** Summary of demographic information, clinical presentation, and therapy about the patients involved in the study.

Patient No.	Gender	Age at Diagnosis (Years)	Location	Symptoms at Onset	Night Pain	Pain with Activities	Relief with NSAIDs	Joint Swelling	Limitation of Motion	Time to Diagnosis (Months)	Initial Misdiagnosis	Therapy
1	F	13	right femoral neck, intra-articular, subperiosteal	pain, anterior thigh	no	no	N/A	-	yes	2	no	CT-guided RFA
2	F	10	right femoral neck, intra-articular, cortical	recurrent pain, right hip and knee	yes	no	yes	-	yes	24	recurrent hip synovitis	N/A
3	M	18	left femoral neck, juxta-articular subcortical	pain, left hip	yes	no	yes	-	yes	5	ileo-psoas enthesopathy	N/A
4	F	15	left acetabulum, intra-articular cortical	pain, left hip	no	yes	no	-	yes	3	no	N/A
5	F	18	right tibia, upper third, juxta-articular subcortical	pain, right knee	no	yes	yes	no	yes	1	arthritis	observation
6	M	17	left tibia, upper third, juxta-articular subcortical	pain, left knee	yes	no	yes	no	no	8	no	CT-guided RFA
7	F	15	right distal fibula, juxta-articular cortical	pain, right knee and leg	yes	yes	yes	no	no	4	amplification pain syndrome	N/A
8	F	14	right humerus, olecranon fossa, intra-articular cortical	pain, right elbow	yes	yes	yes	yes	yes	9	post-traumatic arthritis	CT-guided RFA
9	M	15	right second toe, distal phalanx, juxta-articular cortical	swelling and pain, second toe	yes	no	yes	yes	no	12	psoriatic arthritis	surgery resection
10	F	5	pedicle L1, intra-articular cortical	back pain, scoliosis	yes	yes	no	-	yes	2	traumatic vertebral fracture	surgery resection

N/A, not available; NSAIDs, non-steroidal anti-inflammatory drugs; CT, computed tomography; RFA, radiofrequency ablation.

**Table 2:** Summary of the available literature about intra- and juxta-articular osteoid osteoma.

Study, Year	Sample (Patients ≤ 18 Years Old)	Average Age at Diagnosis (Years)	Location	Night Pain	Relief with NSAIDs	Joint Swelling	Limitation of Motion	Average Time to Diagnosis (Years)	Arthritis as Misdiagnosis
Pikoulas et al., 1995 [3]	6	15	femoral neck (2/6), tibia (1/6), acetabulum (2/6), acromion (1/6)	2/6	5/6	0/6	1/6	1	N/A
Szendroi et al., 2004 [8]	8	15	(1/11), calcaneus (1/11)	1/8	6/8	N/A	8/8	1,2	N/A
Knezevic et al., 2022 [15]	4	13	olecranon fossa (4)	2/4	3/4	2/4	3/4	1,3	3/4
Traore et al., 2014 [16]	4	11	talus (4/8), acetabulum (2/8), femur (2/8)	2/4	1/4	1/4	4/4	0,4	4/4
Albisinni et al., 2014 [17]	4	14	talus (1/4), femoral neck (3/4)	N/A	N/A	N/A	N/A	1,1	N/A
Filippiadis et al., 2017 [18]	6	12	hip (5/6), foot (1/6)	N/A	N/A	N/A	6/6	N/A	N/A
Georgoulis et al., 1995 [19]	9	15	femur (6/9), lumbar spine (1/9), carpus (1/9), patella (1/9)	N/A	9/9	N/A	N/A	1,6	2/9
Payo-Ollero et al., 2019 [20]	5	14	calcaneus (4/5), talus (1/5)	5/5	5/5	0/5	0/5	1,8	1/5
Cassar-Pullicino et al., 1992 [21]	4	13	femur (1/4), talus (1/4), ulna (1/4), MTF (1/4)	N/A	1/4	3/4	4/4	1,3	2/4
Goldberg et al., 1975 [22]	31	N/A	femur (30), acetabulum (1)	19/31	21/31	N/A	25/31	1,7	7/31
May et al., 2019 [23]	50	12	femur (43), acetabulum (7)	45/50	43/50	N/A	17/50	0,6	2/50
Peyser et al., 2009 [24]	5	N/A	femur (3/5), calcaneus (1/5), talus (1/5)	N/A	N/A	N/A	N/A	N/A	N/A
Song et al., 2015 [25]	11	10	femur (8/11), prox humerus (1/11), dist humerus	4/11	4/11	N/A	8/11	1	2/11

N/A, not available; NSAIDs, non-steroidal anti-inflammatory drugs.

## Data Availability

Not applicable.
